# Real-life experience in the treatment of gallbladder carcinoma by transarterial radioembolization: a multicenter retrospective study

**DOI:** 10.1186/s41824-026-00304-9

**Published:** 2026-05-25

**Authors:** Thomas Dertnig, Roman Kloeckner, Lukas Mueller, Timo Alexander Auer, Arthur J. A. T. Braat, Malte Maria Sieren, Ulf Neumann, Stefan Kasper, Leonie S. Jochheim, Benedikt M. Schaarschmidt

**Affiliations:** 1 Department of Diagnostic and Interventional Radiology and Neuroradiology, University Hospital Essen, Hufelandstraße 55, 45147 Essen, Germany; 2 Institute of Interventional Radiology, University Hospital Schleswig-Holstein- Campus Lübeck, 23538 Lübeck, Germany; 3 Department of Diagnostic and Interventional Radiology, University Medical Center of the Johannes Gutenberg-University Mainz, Mainz, Germany; 4 Department of Radiology, Charité-Universitätsmedizin Berlin, Corporate Member of Freie Universität Berlin and Humboldt-Universität zu Berlin, 13353 Berlin, Germany; 5 Department of Radiology and Nuclear Medicine, UMC Utrecht, Utrecht, The Netherlands; 6 Department of Nuclear Medicine, Netherlands Cancer Institute, Amsterdam, the Netherlands; 7 Department of Radiology and Nuclear Medicine, University Hospital Schleswig-Holstein, Ratzeburger Allee 160, 23562 Lübeck, Germany; 8 Department of General, Visceral and Transplant Surgery, University Hospital Essen, Essen, Germany; 9 Department of Medical Oncology, West German Cancer Center, University Hospital Essen, Essen, Germany; 10 Department of Gastroenterology and Hepatology, Essen University Hospital, Essen, Germany; 11 Clinic of Nuclear Medicine, University Hospital Essen, Essen, Germany

**Keywords:** Gallbladder carcinoma, Liver metastases, Radioembolization, Yttrium-90, Interventional oncology

## Abstract

**Purpose:**

Patients with liver metastases from gallbladder carcinoma have limited treatment options. This retrospective multicenter study evaluated overall survival, tumor control and safety after transarterial radioembolization in this rare patient group.

**Materials and methods:**

Patients with histologically confirmed gallbladder carcinoma and liver-dominant metastatic disease treated with yttrium-90 or holmium-166 radioembolization at five tertiary centres were retrospectively identified. Baseline clinical and imaging data, details of the radioembolization procedure and follow-up imaging were collected. Tumor response was assessed locally using RECIST 1.1. Overall survival was analysed descriptively using Kaplan–Meier curves.

**Results:**

Sixteen patients (9 women, mean age 65.7 ± 5.5 years) were included; 93.8% had received prior systemic chemotherapy and 75.0% had extrahepatic metastases. At approximately 3 months, hepatic disease control was achieved in 81.8% (9/11) of patients with available imaging, whereas global disease control was 36.4% (4/11). Median overall survival was 7.1 months (95% confidence interval 1.7–12.3). The median time to hepatic progression was 115 days and the median time to global progression was 112 days. Procedure-related complications were limited to one coil dislocation, one contrast reaction and one liver abscess.

**Conclusion:**

Radioembolization was feasible and well tolerated in patients with liver-dominant metastases from gallbladder carcinoma and provided intrahepatic disease control in a heavily pretreated population. Given the small sample size, these exploratory findings require confirmation in prospective studies.

## Key points

Question:

Can transarterial radioembolization (TARE) be a feasible and safe liver-directed treatment approach in carefully selected patients with advanced gallbladder carcinoma and liver-dominant disease? Pertinent findings:

In this small retrospective multicenter cohort, median overall survival was 7.1 months, hepatic disease control at 3 months was high among evaluable patients, and short-term safety was favorable; however, global disease control was substantially lower due to frequent extrahepatic progression. Implications for patient care:

Radioembolization may be considered in carefully selected patients and in experienced centers as a palliative liver-directed treatment approach when further systemic therapy is not feasible.

## Introduction

Gallbladder carcinoma (GBC) is a rare but highly aggressive malignancy with a dismal prognosis and a 5-year survival rate of only 5–15% (Chang et al. 2020, Coimbara et al. 2020). Although early detection allows curative resection with reported survival rates up to 75%, most patients present with advanced disease and are no longer eligible for surgery (Chang et al. 2020, Coimbra et al. 2020).

Cholecystolithiasis is the most relevant risk factor and is present in up to 90% of patients, while additional risk factors include chronic inflammation, gallbladder polyps, obesity, porcelain gallbladder, primary sclerosing cholangitis, and various infectious or hereditary conditions (Palmer and Patel 2012, Valle et al. 2012, Atchison et al. 2011, Leitlinienprogramm Onkologie 2025, Park et al. 2009, Kratzer et al. 2010, Nagaraja and Eslick 2014, Patel et al. 2011). Polyps ≥ 10 mm, particularly when associated with cholelithiasis, carry a significantly increased risk of malignant transformation (Park et al. 2009).

For advanced or metastatic biliary tract cancers, gemcitabine–cisplatin (GEMCIS) remains the first-line standard. The addition of durvalumab has demonstrated survival benefits in biliary tract cancers overall, although subgroup analyses indicate that patients with GBC benefit less than those with intrahepatic or extrahepatic cholangiocarcinoma (Leitlinienprogramm Onkologie 2025, Oh et al. 2022). In later lines of therapy, options such as FOLFOX or targeted agents (e.g., FGFR and IDH inhibitors) may be available, but actionable molecular alterations are considerably rarer in GBC, limiting the applicability of targeted therapies (Leitlinienprogramm Onkologie 2025).

Local liver-directed therapies for metastatic GBC remain poorly studied. Retrospective data on hepatic arterial infusion chemotherapy (HAIC), alone or combined with PD-1 inhibitors and bevacizumab, have shown encouraging disease control and manageable toxicity in selected patients (Zhao et al. 2024, Zheng et al. 2021).

Transarterial radioembolization (TARE) with Yttrium-90 microspheres is an established treatment modality for primary and secondary liver tumors and has demonstrated promising results in intrahepatic cholangiocarcinoma in multicenter retrospective studies (Schaarschmidt et al. 2022, Schaarschmidt et al. 2023, Edeline et al. 2020, Bargellini et al. 2020). However, evidence regarding its role specifically in metastatic GBC is scarce.

Therefore, the objective of this multicenter retrospective study was to explore the feasibility, safety, overall survival, and tumor control of TARE in carefully selected patients with metastatic GBC and liver-dominant disease.

## Materials and methods

We conducted a retrospective analysis of patients with GBC who underwent TARE at five tertiary care centers in Germany and the Netherlands between January 2015 and December 2021. Eligible patients had a confirmed diagnosis of adenocarcinoma of the gallbladder and received either ⁹⁰Y or ¹⁶⁶Ho TARE. Patients with intra- or extrahepatic cholangiocarcinomas, including Klatskin tumors, were excluded from the study.

Data collection was standardized across centers using a uniform questionnaire. All patient data were anonymized and compiled into a central database for statistical analysis. The study was approved by an institutional ethics committee (application number: 20-9747-BO).

### Data collection

Patient demographics, clinical history, tumor characteristics, prior systemic treatments, laboratory parameters, biliary interventions, imaging findings and details of the radioembolization procedure were retrospectively collected from electronic medical records. Baseline tumor burden, presence and sites of extrahepatic disease, liver function tests and performance status were recorded.

Follow-up imaging was performed according to institutional standards, typically using contrast-enhanced CT or MRI at approximately 3 and 6 months after treatment. Tumor response was assessed locally using RECIST 1.1. All recorded variables are summarized in Tables 1 and 2.

**Table 1:** Baseline characteristic

Patient characteristics	Category 1	Category 2	%	*n*
Sex	female		56.3	9
	male		43.7	7
Median Height	171.8 ± 10.1 cm			
Median Weight	78.8 ± 23.8 kg			
Mean age at the MAA	65.7 ± 5.5 years females: 63.9 years males: 68.5 years			
Initial T-Stage	2		6.25	1
	3		31.25	5
	4		43.75	7
	Relapse		12.5	2
	Unknown		6.25	1
Initial N-Stage	0		18.75	3
	1		68.75	11
	Unknown		12.5	2
Initial M-Stage	0		43.75	7
	1		56.25	9
Initial tumor grading	G2		37.5	6
	G3		43.75	7
	G4		6.25	1
	Unknown		12.5	2
Initial UICC tumor stage	IVa		43.75	7
	IVb		56.25	9
Extrahepatic metastases at the time of TARE	Yes		75	12
	No		25	4
Prior Therapy	Yes		100	16
		Hepatic surgery	68.75	11
		Radiation	6.25	1
		Local ablation (MWA/RFA)	6.25	1
		Chemotherapy	93.75	15
		Antibody Therapy	6.25	1
Vascularisation pattern		Hypervascularized	31.25	5
		Hypovascularized	50	8
		Mixed	18.75	3
Tumor type		Mass-forming	36.4	4
		Diffuse	63.6	7
Number of lesions		1	9.1	1
		< 3	27.3	3
		< 10	36.4	4
		> 10	27.3	3
Hepatic tumor burden		< 25%	81.25	13
		25–50%	18.75	3

**Table 2:** Best overall hepatic response (HR) and global response (GR) according to RECIST 1.1 criteria, along with time to hepatic progression (TTHP), time to global progression (TTGP), and overall survival (OS) in days for each patient

Patient	HR	GR	TTHP [d]	TTGP [d]	OS [d]
1	PR	PR	98	98	98
2	SD	PD	115	119	119
3	SD	SD	128	128	128
4	-	-	20	20	20
5	-	-	137	137	137
6	SD	PD	157	58	249
7	PD	PD	112	112	280
8	-	-	111	111	111
9	-	-	193	216	216
10	SD	PD	88	69	88
11	SD	PD	86	64	86
12	SD	SD	236	133	236
13	-	-	51	51	51
14	SD	SD	50	50	50
15	PD	PD	92	92	92
16	SD	PD	90	90	298

“-” indicates that no response evaluation was available

### Statistical analysis

Owing to the retrospective design and small sample size, all analyses were descriptive. Continuous variables were summarized as mean ± standard deviation or median with range, as appropriate, and categorical variables as absolute and relative frequencies. Overall survival was illustrated using Kaplan–Meier curves without statistical comparison or hypothesis testing.

Tumor response at follow-up was analyzed in a response evaluable set, defined as all patients with available cross-sectional imaging at the respective follow-up time point and assessed locally according to RECIST 1.1 criteria. In addition, an intention-to-treat sensitivity analysis was performed for disease control at 3 months, in which all 16 treated patients were included and patients without available follow-up imaging were conservatively imputed as having progressive disease. Statistical analyses were performed using IBM SPSS Statistics Version 30 (IBM, Armonk, NY, USA).

## Results

### Baseline characteristics

Datasets of 16 TARE procedures from 16 individual patients were collected from five tertiary care centers across two countries (Germany and the Netherlands). The patient cohort included 9 females (56.3%) and 7 males (43.7%). The mean age at the time of the MAA scan was 65.7 ± 5.5 years (females: 63.9 years; males: 68.5 years). Extrahepatic metastases were documented in 75% (12/16). The baseline characteristics are shown in Table 1.

In total, 93.8% (15/16) of patients had received prior oncological treatments including surgery (*n* = 11), chemotherapy (*n* = 15), radiation therapy (*n* = 1), local ablation (*n* = 1), and antibody therapy (*n* = 1). Progressive disease (PD) was noted as the response to prior therapy in 68.8% (11/16) before TARE.

**Fig. 1:**
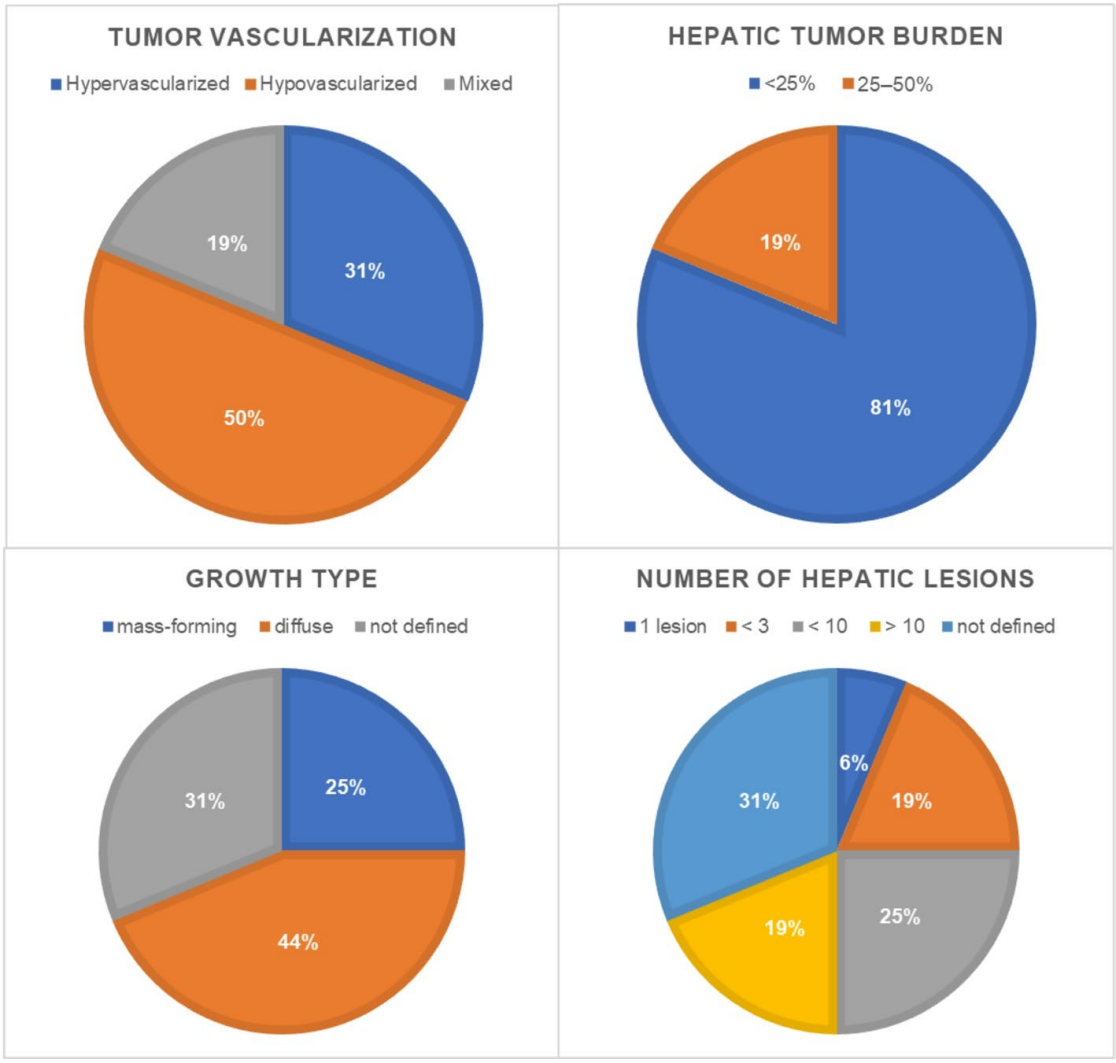
Tumor characteristics of the study cohort. Pie charts show the distribution of (**A**) tumor vascularization (hypervascularized, hypovascularized, or mixed), (**B**) hepatic tumor burden (< 25% vs. 25–50%), (**C**) tumor growth type (mass-forming, diffuse, or not defined), and (**D**) number of hepatic lesions (1 lesion, < 3, <10, > 10, or not defined). Percentages indicate the proportion of patients in each category

Tumor vascularization was described as hypervascularized in 31.3% (5/16) of cases, hypovascularized in 50.0% (8/16), and mixed in 18.8% (3/16). A mass-forming tumor type was identified in 25.0% (4/16) of patients, while diffuse intrahepatic tumor growth was observed in 43.8% (7/16). Tumor burden was < 25% in 81.3% (13/16) of cases and between 25 and 50% in 18.8% (3/16). Partial portal vein thrombosis was present in 6.3% (1/16) of patients. The number of hepatic lesions varied: 6.3% (1/16) had 1 lesion, 18.8% (3/16) had fewer than 3 lesions, 25.0% (4/16) had fewer than 10 lesions, and 18.8% (3/16) had more than 10 lesions. The distribution of tumor vascularization, growth pattern, tumor burden, portal vein thrombosis, and number of hepatic lesions among the study cohort is summarized in Fig. 1.

### Preinterventional evaluation and radioembolization

Preinterventional imaging was performed using CT in 87.5% (14/16), MRI in 18.8% (3/16), and both imaging modalities in 6.3% (1/16). Prior to selective MAA injection, coiling was necessary in 50.0% (8/16). Preinterventional MAA scintigraphy revealed a mean lung shunt fraction of 4.1 ± 2.1%. In 6.3% (1/16), the lung shunt fraction was > 10%.

TARE was performed within a mean time span of 22.6 ± 14.9 days after MAA injection. Unilobar treatment was performed in 43.8% (7/16), bilobar in 50.0% (8/16), and central segment treatment in 6.3% (1/16). A biliary stent was present in 31.3% of patients (5/16), while 68.8% (11/16) had no biliary stent in place. ^90^Y Glass microspheres (TheraSphere^®^) were used in 56.3% (9/16), ^90^Y resin microspheres (SIR-Spheres^®^) in 37.5% (6/16), and ^166^Ho radioembolization in 6.3% (1/16).

The calculated ^90^Y activity was 1.5 ± 0.46 GBq, while 6.1 GBq was administered in the ^166^Ho case.

Peri- and postinterventional complications occurred in 18.8% (3/16), including coil dislocation (*n* = 1), allergic reaction to contrast agent (*n* = 1), and liver abscess (*n* = 1).

### Follow-up examinations

Follow-up examinations were available at approximately 3 months for 11 of 16 patients (mean: 82 ± 27.5 days) and at approximately 6 months for 3 patients (mean: 153 ± 15 days). In the response evaluable set at 3 months (*n* = 11), the global disease control rate (DCR), defined as partial response (PR) or stable disease (SD), was 36.4% (4/11). Hepatic disease control was achieved in 81.8% (9/11), with stable disease in 72.7% (8/11) and partial response in 9.1% (1/11), as shown in Table 2. Hepatic progression occurred in 18.2% (2/11), while extrahepatic progression was observed in 54.5% (6/11).

In the predefined worst-case intention-to-treat sensitivity analysis including all 16 treated patients, patients without follow-up imaging were imputed as having progressive disease, resulting in a global DCR of 25.0% (4/16).

### Survival analysis

**Fig. 2:**
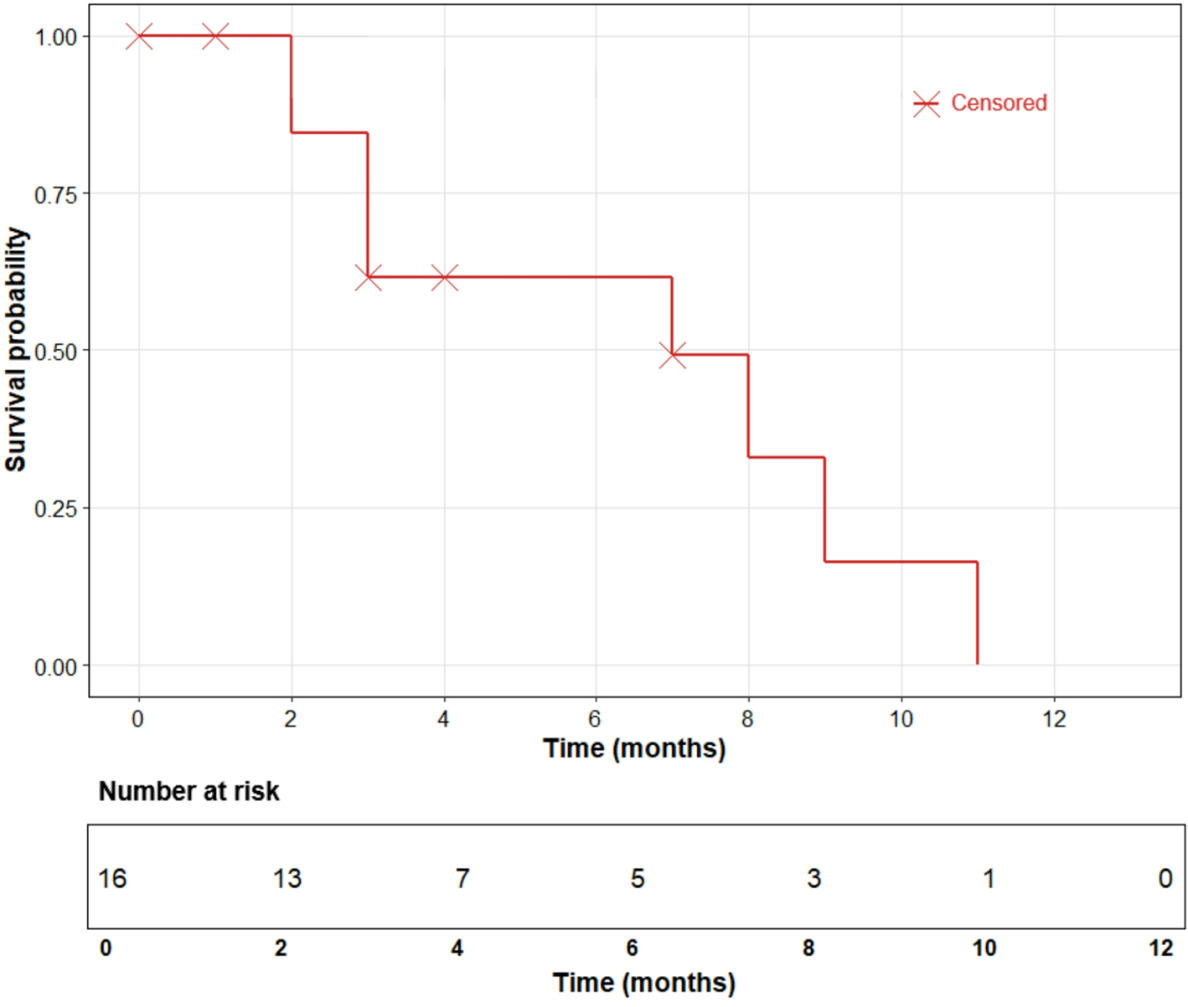
Median OS in months. Kaplan-Meier curve showing overall survival. Censored observations are indicated by red “×” marks. The x-axis denotes time in months. The table below the plot presents the number of patients at risk at each time point

The median OS for the entire cohort was 7.1 months, with a 95% confidence interval (CI) ranging from 1.7 to 12.3 months (Fig. 2).

**Fig. 3:**
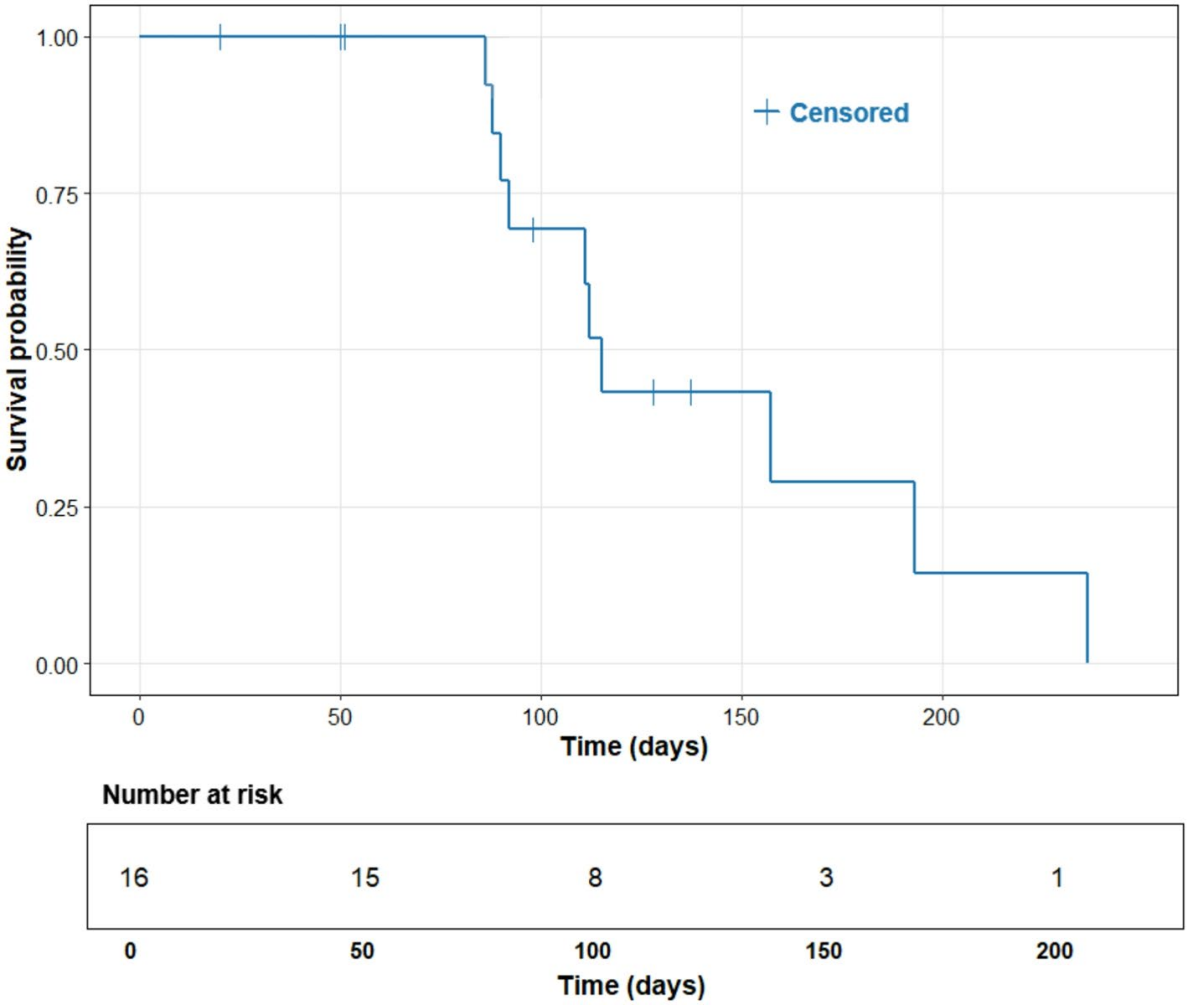
Kaplan–Meier curve for time to hepatic progression (TTHP) in days. The blue line represents the cumulative survival function, with crosses denoting censored cases. The number at risk is displayed below the x-axis for corresponding time intervals

**Fig. 4:**
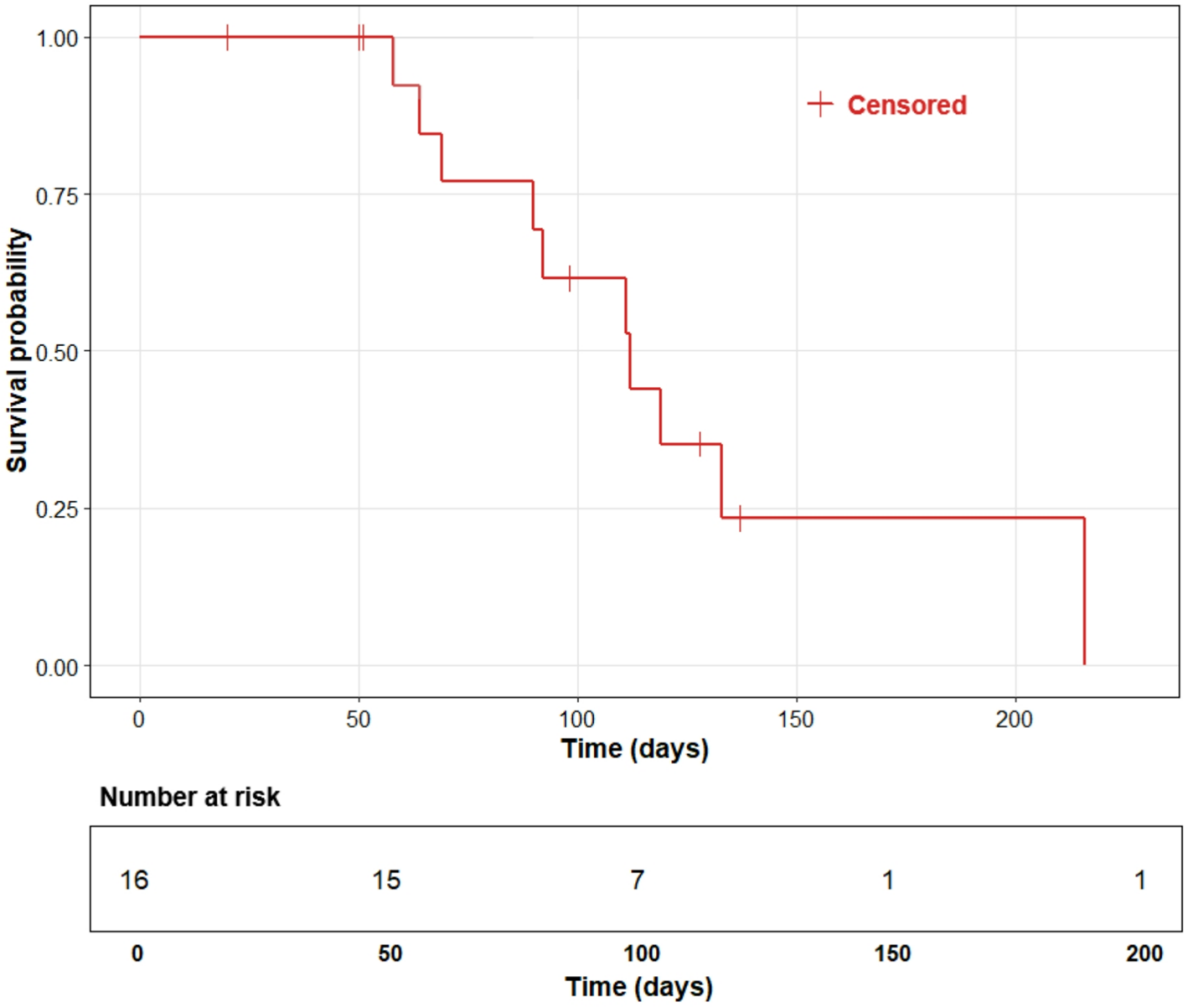
Median time to global progression. Kaplan–Meier curve illustrating time to global progression (TTGP) in days. Censored observations are marked with red crosses. The number at risk is shown below the x-axis

The median time to hepatic progression, defined as the time from treatment to radiologically confirmed progression of intrahepatic disease, was 115 days (95% CI: 108.5 to 121.5 days) (Fig. 3). Similarly, the median time to global progression, which includes both hepatic and extrahepatic progression as well as death, was 112 days (95% CI: 80.2 to 142.8 days) (Fig. 4).

## Discussion

In this multicenter cohort of 16 patients treated with TARE for metastatic GBC, most patients had extrahepatic disease (75%) and extensive prior therapy (94%). Median OS was 7.1 months, while median hepatic and global progression occurred at 115 and 112 days, respectively. Hepatic disease control was achieved in most evaluable patients, but global DCR was markedly lower due to frequent extrahepatic progression, underscoring the systemic aggressiveness of GBC. These findings should be interpreted as exploratory only and should not be considered proof of a survival benefit, but rather as a feasibility and safety signal in a highly selected population with liver-dominant disease treated at experienced tertiary centers.

Comparable data for liver-directed therapies in GBC remain scarce. Retrospective studies on HAIC, alone or combined with immunotherapy, have shown encouraging disease control in selected patients (Zhao et al. 2024, Zheng et al. 2021). Evidence from TARE in intrahepatic cholangiocarcinoma suggests favorable intrahepatic tumor control and acceptable toxicity (Schaarschmidt et al. 2023), which aligns with the high hepatic DCR observed here. However, the predominance of extrahepatic progression in our cohort limits the overall impact of liver-directed therapy on global tumor burden.

Systemic options beyond first-line GEMCIS ± immunotherapy provide only modest benefit, with median OS around 4–8 months in second-line settings (Goldstein et al. 2011, Lee et al. 2008, Doval et al. 2004, Harder et al. 2006, Sharma et al. 2016). Many GBC patients cannot tolerate further chemotherapy due to comorbidities, ECOG ≥ 2, or biliary complications. In this context, TARE may represent a potential palliative liver-directed option with minimal systemic toxicity and outpatient feasibility in carefully selected patients, particularly when further systemic treatment is not feasible. Median OS in our cohort exceeded historical outcomes for best supportive care (3.25–4.5 months) (Sharma et al. 2016, Ji et al. 2015, Singh et al. 2018), however, given the retrospective design, very small sample size, and likely selection bias, this observation should be interpreted as hypothesis-generating rather than comparative evidence.

Safety outcomes were favorable, with only three low-grade complications (coil dislocation, contrast reaction, abscess) and no procedure-related mortality. No patient developed new-onset jaundice attributable to TARE, which is clinically relevant given the frequency of biliary obstruction in GBC.

Several limitations must be acknowledged. The small sample size and retrospective multicenter design limit statistical precision and may introduce heterogeneity and selection bias. Five patients lacked follow-up imaging, necessitating a worst case sensitivity analysis for disease control, which reduced global DCR to 25%. Response assessment was based on local RECIST reads without centralized review, introducing inter-reader variability. Imaging was predominantly CT-based, which may underestimate tumor burden compared with MRI. Finally, personalised dosimetry could not be analysed due to inconsistent availability, although it may be relevant for outcomes in tumors with heterogeneous perfusion.

Despite these limitations, our findings provide preliminary real-world evidence that TARE is technically feasible and appears safe in carefully selected patients with metastatic GBC and liver-dominant disease. Larger prospective studies are needed to better define patient selection criteria, to evaluate the role of standardized imaging and dosimetry, and to clarify how TARE may be integrated with contemporary systemic treatment strategies.

## Conclusion

This study suggests that radioembolization is a feasible and well-tolerated liver-directed treatment approach in carefully selected patients with metastatic gallbladder carcinoma and liver-dominant disease when systemic options are limited. Given the small sample size and retrospective design, these results are exploratory and should not be interpreted as evidence of a survival benefit, but rather as a rationale for prospective evaluation.
